# Dynamic pricing with demand disaggregation for hotel revenue management

**DOI:** 10.1007/s10732-021-09480-2

**Published:** 2021-06-07

**Authors:** Andrei M. Bandalouski, Natalja G. Egorova, Mikhail Y. Kovalyov, Erwin Pesch, S. Armagan Tarim

**Affiliations:** 1 United Institute of Informatics Problems, National Academy of Sciences of Belarus, Minsk, Belarus; 2 Institute of Information Systems, Faculty III, University of Siegen, Siegen, Germany; 3 Center for Advanced Studies in Management, HHL Leipzig, 04109 Leipzig, Germany; 4 Cork University Business School, University College Cork, Cork, Ireland

**Keywords:** Hotel revenue management, COVID-19, Dynamic pricing, Demand elasticity, Concave programming

## Abstract

In this paper we present a novel approach to the dynamic pricing problem for hotel businesses. It includes disaggregation of the demand into several categories, forecasting, elastic demand simulation, and a mathematical programming model with concave quadratic objective function and linear constraints for dynamic price optimization. The approach is computationally efficient and easy to implement. In computer experiments with a hotel data set, the hotel revenue is increased by about 6% on average in comparison with the actual revenue gained in a past period, where the fixed price policy was employed, subject to an assumption that the demand can deviate from the suggested elastic model. The approach and the developed software can be a useful tool for small hotels recovering from the economic consequences of the COVID-19 pandemic.

## Introduction

A rapid development in the fields of information technologies and e-commerce has supported the use of dynamic pricing approaches in hotels and the research interest in this area. The fact that the absolute majority of reservations come from the Internet makes it easy to simulate future demand in all categories, dynamically calculate and offer appropriate prices, and automatically link every incoming reservation to the appropriate category. For example, practical hotel revenue management (HRM) systems, that use dynamic pricing, are described by Koushik et al. (2012) and Pekgun et al. (2013). However, the practical systems we know are expensive software tools, used by the major hotel chains. Many small hotels do not consider acquisition of a commercial HRM system as profitable.

In this paper we describe a general HRM problem and a solution approach that is easy to implement, yet showing good performance in the experiments with data sets of a small size hotel. The source code of the developed software and the results of the experiments can be downloaded from Bandalouski et al. (2020). The software and the materials in this paper provide an opportunity for small hotels to use optimization techniques to aid their recovery from the economic consequences of the COVID-19 lockdown.

The World Tourism Organization UNWTO (2020) reports a decrease of 22% in tourist arrivals in the first quarter of 2020, and considers three scenarios with declines of 58%, 70% and 78% in international tourist arrivals for the whole year. The essential recovery is supposed to start in 2021. The International Association of Scientific Experts in Tourism (AIEST) AIEST (2020) discusses solutions to limit the economic impact of the COVID-19 lockdown. The solutions include redesign of business processes and changing services to avoid sharing space. A new important business criterion is to maximize revenue per square unit per unit of time. A suitable electronic reservation tool is required to meet this criterion.

Our approach to the HRM problem includes the determination of input parameters for the subsequent mathematical analysis, disaggregation of the demand into several categories, demand forecasting for the *reference price*, simulation of the demand-price relations, and a mathematical programming model for dynamic price optimization. A reference (or *base*) price for a demand category is the price of the last sale in this category, which can periodically be adjusted to the prices of competitors. The decision variables are prices for rooms in each demand category and on each day of the planning horizon. The day is assumed to be the time between the check-in time and the check-out time with one night between them. The introduction of low and high season categories allows to resolve the seasonality issue and takes into account the demand for special events. The demand forecast, the simulation of the demand-price relations and the determination of the optimal prices are carried out separately for each category and each day of the planning horizon in order to more easily understand, model and solve the complex problem. Prices are variables in the mathematical programming model, the criterion of which is to maximize the total hotel revenue in the specified period, subject to the limited number of free rooms, the upper and lower price limits and the price hierarchy. The objective function is quadratic as it results from the multiplication of the prices and the corresponding demand values depending on the price. The constraints are linear, so the overall problem is computationally simple. Its solution requires less than a second on a personal computer.

Consider a small size hotel, denoted as ${{\mathcal {H}}}$. In order to make our approach computationally efficient and easy to implement, we accept the following realistic assumptions: (1) disaggregation of the demand into categories has a positive effect on the accuracy of the demand forecast, (2) demands of different categories do not correlate with each other, (3) past demand is not constrained (by the available rooms), that is, either it was always completely satisfied or, if this was not the case then the sum of the unsatisfied and satisfied room requests is used as the past demand, (4) demand is *elastic*, that is, it is a linear non-increasing function of the price, (5) the slope of the demand function depends solely on the demand category, (6) the effect of causal variables, other than the prices of hotel ${{\mathcal {H}}}$, on the slope of the demand function is minor such that it can be acceptably estimated by a simple linear regression model based on the past demand and corresponding price values of the hotel ${{\mathcal {H}}}$, (7) the demand for the reference price can be acceptably estimated by time series methods in which no causal variable is included, (8) the constant of the demand function depends on the demand category and the day of stay, and can be calculated by using the reference price, the slope of the demand function and the demand value forecasted for the reference price, (9) the effect of causal variables other than the prices of hotel ${{\mathcal {H}}}$, such as prices of competitors, on future demand can be accounted by a periodical adjustment of the reference price by the managers.

Revenue management approaches first appeared in the passenger air traffic in the late 1970s and are currently well developed there. Numerous results are obtained for seat allocation, demand forecasting and pricing, with or without competition, see, for example, Currie et al. (2008). Techniques such as linear and nonlinear programming (Weatherford 2001; Talluri and van Ryzin 1999), dynamic programming (You 1999; Bitran and Monschein 1995) and stochastic programming (van Ryzin and Talluri 2003) were applied for seats allocation problems. You (1999) and van Ryzin and Gallego (1997) developed approaches that combine pricing and resource management. Historical, advanced and combined forecasting methods were used to predict passengers’ demand (Lee 1990). In the 1990s, other companies, including the hotel business, began to gain experience in revenue management by adapting its principles, models and tools to their own specifics, see, for example, Xiong and Li (2013) and Chung and Li (2014). In 2005 Talluri and van Ryzin (2005) gave a comprehensive review of the revenue management research, including a part devoted to the hotel industry. Recent reviews of literature on hotel revenue management are given by Guillet and Mohammed (2015), Bandalouski et al. (2018), Vives et al. (2018) and Gönsch (2020). The most recent publications on this topic include Chen et al. (2017), Cui et al. (2018) and Gallego and Topaloglu (2019).

The rest of the paper is organized as follows. The next section describes a general scheme of our approach. The mechanism of demand disaggregation and input parameters for further mathematical analysis are discussed in Sect. 3. Forecasting techniques are presented in Sect. 4. Section 5 deals with the determination of demand-price relations. An optimization model is given in Sect. 6. While the optimization model assumes an exact solution, it is developed based on heuristic reasoning, therefore, our approach to the hotel dynamic pricing is heuristic in general. Computer experiments are described in Sect. 7. The paper concludes with a short summary of the results and suggestions for future research.

## General scheme

Next we discuss the main steps for successfully using our dynamic pricing approach. A client who books a room via a booking portal specifies some attributes, which are mainly time and length of her stay and possibly further information about the room type (e.g. room size, shower or bath, balcony, kitchen, etc.). Rooms of the same type can be offered at different room prices (tariff classes), which are characterized by additional services (free toiletries, welcome food and drinks, room service, satellite TV channels, Internet access, breakfast included, etc.). Some rooms allowing multiple tariff classes can be converted from one tariff class to another. Then we address the four main steps:

1. Specification of the following input data:
  The demand categories defined by attributes like the time of the booking, time and length of the stay, and the room type defined by the tariff class (cf. Sect. 3), and
  For each category its reference price (with its lower and upper limits as 50% deviations),
  The operational cost for each room type and the
  Planning horizon.
2. Forecasting the demand for the reference price (cf. Sect. 4), based on
  Historical data on the check-ins and associated lengths of stay for each demand category in order to
  Forecast for each demand category the number of check-ins and length of stay for each day of the planning horizon, which allows a
  Calculation of the number of occupied rooms for each demand category and day in the planning horizon.
3. Determination of the demand-price relations as linear non-increasing demand functions for each category (cf. Sect. 5) which means the
  Calculation of the slope of the function by a linear regression based on the past demand-price values, and the
  Calculation of the constant of the demand function using the reference price, the slope of the demand function and the forecasted demand for the reference price.
4. Calculating an optimal price $p_{\tau ,c}$ for each category *c* and each day $\tau$ in the planning horizon by solving a mathematical programming problem with a concave quadratic objective function and linear constraints on the price values (cf. Sect. 6).

Steps 2–4 are applied repeatedly after every registered booking request. Below we describe our dynamic pricing approach in more detail.

## Demand disaggregation and determination of input parameters and decision variables

We suggest that the demand is disaggregated into several categories, characterized by a set of parameters. Booking requests with the same set of these parameters comprise the same demand category. Computer experiments conducted by Weatherford et al. (2001) have shown that demand disaggregation provides a higher level of forecasting accuracy. We have found that, while demand disaggregation facilitates solving the succeeding optimization problem, it can reduce the quality of the forecast by making the data of the demand time series extremely sparse and disrupting the regularity of the data. Therefore, there should be a reasonable balance between the number of categories and the scarcity of data in the categories.

We will illustrate our approach by considering the example hotel ${{\mathcal {H}}}$. A historical database over four years of daily bookings is available for this hotel. We divide the demand of hotel ${{\mathcal {H}}}$ into categories according to the following parameters:

- Type of season denoted as *Season*,
- Day of the week (*Day*),
- Length of stay (*Length*),
- Length of the time period between the time of reservation and the check-in time (*Before*), and
- Tariff class (*Tariff*).

For hotel ${{\mathcal {H}}}$, we differentiate between two seasons: the low season (*Low*) includes January–March and November, and the high season (*High*) includes the rest of the year. No special events have occurred in the past and are not expected in the future. Days of the week are characterized as weekdays (*Mon–Thu*) that include Monday to Thursday, and weekends (*Fri–Sun*) with Friday to Sunday. Demand of the same type of season and day of the week has no seasonal and weekly fluctuations. With the introduction of the parameters *Season* and *Day* we no longer have to “clean” the data from seasonality in the forecast stage.

We assume that the length of stay can be of two classes: less than or equal to 7 days ($7-$) and more than 7 days ($8+$). The length of the time period between reservation and check-in can be less than or equal to 7 days ($7-$), 8–30 days (8–30) and more than 30 days ($31+$). The approach does not set a price for spot demand, that is, for check-ins without a reservation. We also define five tariff classes: economy class (*E*), premium economy class (*E+*), business class (*B*), premium business class (*B+*) and suite (*S*), which are partitioned into $r=3$ *tariff-convertible* types: type 1 includes economy class and premium economy class, type 2 includes business class and premium business class, and type 3 includes suite only. A room of a certain tariff-convertible type can be converted from one tariff class to another tariff class of the same type at no cost.

The result of the above classification is that each reservation is assigned to one of the 120 demand categories. The value 120 is obtained by multiplying numbers of values of the category parameters: $120 = 2 \times 2 \times 2 \times 3 \times 5$. Demand categories are denoted as *c*, $c=1,\ldots ,k$, where $k=120$ for the hotel ${{\mathcal {H}}}$. Demand category *c* is represented as follows:

$$\begin{aligned} c=(Season=X, Day=Y, Length=Z, Before=V, Tariff=W), \end{aligned}$$

where $$X\in \{Low, High\}$$, $$Y\in \{Mon{-}Thu, Fri{-}Sun\}$$, $$Z\in \{7-, 8+\}$$, $$V\in \{7-,8{-}30,31+\}$$, $$W\in \{E, E+, B, B+, S\}$$. For example, $$c=(Season=High, Day=Fri$$–$$Sun, Length=7-, Before=7-, Tariff=B)$$. Note that check-in date of a reservation uniquely defines type of the season and day of the week. Denote by $$C_\tau $$ a subset of *eligible* categories which are uniquely defined by the check-in day $$\tau $$.

Denote by $M_i$ the set of categories with rooms of the tariff-convertible type *i*, and assume that categories of the same tariff-convertible type are numbered consecutively such that $M_i=\{c_{i-1}+1,c_{i-1}+2,\ldots ,c_i\}$, $i=1,\ldots ,r$, where $c_0:=0$ and $c_r=k$. Assume that there is a requirement of *price hierarchy* between the demand categories of the same tariff-convertible type such that the following relations must be satisfied: $p_{\tau ,c}\le p_{\tau ,c+1}$ for any $c\in C_\tau \cap M_i$ and $c+1\in C_\tau \cap M_i$, $i=1,\ldots ,r$.

The other input parameters for our revenue management approach are the *planning horizon*, the room operational cost for each tariff class, the lower and upper bounds on the price values in the demand categories and the reference price for each demand category. The planning horizon is the period of time in the future for each day of which, starting from today, optimal prices have to be determined. The room operational cost is the per day cost applied if the room is in use. We require that the room price is not lower than the room operational cost.

When adjusting the reference prices, managers can group categories, define the reference price for one category of each group, and then set the percentage deviation from this price for reference prices of other categories of the same group. The reference price setting category can be defined by the highest price. For the hotel $${{\mathcal {H}}}$$, rooms are sold at the highest price in the category $$(Season=High,Day=Fri$$–$$Sun, Length=7-, Before=7-, Tariff=W)$$ of the group of categories with the same tariff class *W*.

The decision variables are prices for rooms for each day $\tau$ of the planning horizon in each demand category from the set $C_\tau$. Note that the day prices for a long stay are not required to be the same, they can be different for different days.

## Demand forecasting for the reference price

We assume that the demand for the reference price can be acceptably estimated by time series methods. It is important to choose a method which guarantees appropriate forecast accuracy. The choice of the forecasting method strongly depends on the historical data at hand and the specificity of the entire HRM approach. In the literature, three major types of time series methods for demand forecasting have been studied: historical, advanced and combined, see, for example, Lee (1990), Rajopadhye et al. (2001) and Ivanov and Zhechev (2012). Disaggregation of the demand into categories may cause an excessive data sparseness. Advanced and combined methods aim at monitoring and maintaining regularities in sparse time series. However, they are sensitive to the input historical data, which affects their quality. Historical methods do not include specific tools to account for data regularities but they are not so sensitive to the input data and provide stable and sufficiently accurate forecast results either for sparse or well saturated time series (Makridakis et al. 1982; Schnaars 1984; Weatherford and Kimes 2003). We employ historical forecasting methods because they are simple and effective with respect to the demand disaggregation.

Demand forecasting starts with building two historical time series for each demand category and day: (1) the number of realized check-ins and (2) the length of stay associated with each check-in. Then, the forecasted numbers of future check-ins and their lengths of stay are linked together in order to calculate the predicted number of occupied rooms in each demand category and day in the planning horizon. These numbers are obtained by means of straightforward arithmetic manipulations with the forecasted check-ins and lengths of stay. The succeeding stages of our HRM approach use the forecasted number of occupied rooms for each category and day.

The length of the planning horizon varies from 1 day to 12 months. The same forecasting method cannot give accurate results for short-term and long-term forecasting horizons. The same observation holds with respect to the data sparseness, which depends on the level of demand disaggregation. We consider demand data of each category as sparse if in at least one of the days of demand time series in the historical period there were no check-ins. For periods of planning up to three months, we employ either *modified Holt’s double exponential smoothing (M-Holt)* or *modified moving average (M-Moving)* methods. This alternative arises due to the density condition of disaggregated historical data. Sparse data leads to inaccurate estimates of the smoothing coefficients of level and trend and, consequently, to an error in the M-Holt forecasting method. Therefore, M-Moving method is applied to sparse data. Since the accuracy of the demand time series extrapolation decreases with the extension of the planning horizon, the approach applies *modified “same day last year” (M-Same)* forecasting method for long-term periods of three months or more. All three forecasting methods are modified by us in order to improve forecast accuracy in the specific environment of our HRM approach.

Let us give details of the forecasting methods. First note that the length of the historical period is method specific. Given a demand category, denote the realized value of the number of check-ins in a past day *i* as $s_i$, $i=1,\ldots ,t$, where *t* is the last day of the historical period, and denote future number of check-ins in a future day *i* as ${\hat{s}}_i$, $i=t+1,\ldots ,t+T$, where *T* is the length of the planning horizon. We assume that check-ins are indexed $1,\ldots ,q,q+1,\ldots ,q+Q$, where 1 is the first (oldest) check-in of the historical period, *q* is the last check-in of the historical period and *Q* is the forecasted number of check-ins in the planning horizon. We have $Q=\sum _{m=1}^T{\hat{s}}_{t+m}$.

We will describe forecasting methods on the example of forecasting the number of check-ins.

**Modified Holt’s double exponential smoothing (M-Holt)**. Holt’s double exponential smoothing forecasting method is an extension of the simple exponential smoothing method, see Gardner (2006) and Rajopadhye et al. (2001). The advantage of Holt’s method is that, besides the smoothed value of the series, it is able to capture medium-term trend. The trend represents the direction in which the series is moving and reflects both internal effects of changes in the hotel as well as external, which affect businesses in the region. The method consists of forecast equation and two smoothing equations:

1 $$\begin{aligned}&{\hat{s}}_{t+m}=l_t+mr_t,\quad m=1,\ldots ,T, \end{aligned}$$

2 $$\begin{aligned}&l_i=\alpha s_i+(1-\alpha )(l_{i-1}+r_{i-1}), \quad i=2,3,\ldots ,t \end{aligned}$$

3 $$\begin{aligned}&r_i=\gamma (l_{i}-l_{i-1})+(1-\gamma )r_{i-1},\quad i=2,3,\ldots ,t \end{aligned}$$

where $l_i$ is an estimate of the level of the series at time *i*, $r_i$ is an estimate of the trend of the series at time *i*. Initial values of $l_1$ and $r_1$ have to be specified. $\alpha$ is a smoothing coefficient for the level, and $\gamma$ is a smoothing coefficient for the trend, $0\le \alpha \le 1$, $0\le \gamma \le 1$.

Equation (1) shows that *m*-step-ahead forecast is equal to the estimated level at day *t* plus *m* times the estimated trend value at day *t*. The Eq. (2) shows that $l_i$ is a weighted average of observation $s_i$ and the within-historical-period one-step-ahead forecast for time *i* given by $l_{i-1}+r_{i-1}$. The trend Eq. (3) shows that $r_i$ is a weighted average of $l_i-l_{i-1}$ and the previous estimate of the trend $r_{i-1}$.

Optimal values for coefficients $\alpha$ and $\gamma$ are estimated from the historical period. Coefficients are estimated by minimizing the *mean squared error (MSE)*. The within-historical-period one-step-ahead forecast errors are specified as $e_i=s_i-{\hat{s}}_i$, $i=1,\ldots ,t$, and MSE is specified as $MSE=\frac{\sum ^t_{i=1} e^2_i}{t}$. To calculate ${\hat{s}}_i$, ${\hat{s}}_i=l_{i-1}+r_{i-1}$, $i=1\ldots t$, initial values of level and trend can be specified as $l_1=s_1$ and $r_1=\frac{(s_2-s_1)+(s_3-s_2)+(s_4-s_3)}{3}$ and initial values of coefficients $\alpha$ and $\gamma$ take arbitrary values within the interval [0,1]. Experiments conducted on the historical data of the hotel ${{\mathcal {H}}}$ showed that the length of the historical period equal to three months gives the best accuracy of the M-Holt forecasting method.

Note that the original method can output fractional parts of the demand values, whose rounding can substantially distort the forecast results. To account for the contribution of the fractional values of check-in time series we suggest to accumulate them starting from the first day of the planning horizon $t+1$. As soon as the sum of the fractional parts of the numbers of check-ins exceeds 1, an extra check-in unit is generated. This unit is randomly added to one of the corresponding days. Therefore, no forecasted demand is lost, which is essential for the disaggregated demand.

**Modified Moving average (M-Moving)**. We use this method for short-term and medium-term planning horizons with up to three months length. Forecast of values of time series for day $t+1$ is calculated by averaging *N*, $1\le N\le t$, previous observations of the series by the following formula:

$$\begin{aligned} {\hat{s}}_{t+1}=\frac{1}{N}\sum ^t_{i=t-N+1}s_i. \end{aligned}$$

The method suggests that ${\hat{s}}_{t+m}={\hat{s}}_{t+1}$, $m=2,3,\ldots ,T$.

The criterion for choosing the optimal number of observations *N* is the smallest forecasting error. Computer experiments on the historical data set of hotel ${{\mathcal {H}}}$ have shown that the minimum forecasting error is attained for $N = 8$. Our modification of the moving average method concerns fractional values. It is similar to that in the M-Holt method.

**Modified method “the same day last year” (M-Same)**. In the forecasting method “the same day last year”, the forecasted value of time series for the demand category in day $t+1$ is assigned the number of check-ins of the corresponding category in the same day of the last year. For example, if there were 77 check-ins on Friday of week 48 of last year, then, according to the method, on Friday of week 48 of this year there will be the same number of check-ins.

To increase the accuracy and add dynamics to the forecasting method “the same day last year”, we suggest to modify it as follows. Let us forecast the number of check-ins for a day $t+1$. Without loss of generality, let this day be Friday. The method M-Same takes the number of check-ins of the same day in the last year but adds to it an average value of deviations of that value from the numbers of check-ins of all Fridays in the last month. If the number of check-ins on Friday of week 48 of last year was 23 and the numbers of check-ins on Fridays of weeks 47, 46, 45 and 44 of this year were 25, 26, 23 and 24, respectively, then the forecasted number of check-ins for Friday of week 48 of this year will be $24,5=23+\frac{2+3+0+1}{4}$. Accounting for the fractional values of check-ins is similar to that in the M-Holt and M-Moving methods.

We conducted computer experiments with the data of hotel ${{\mathcal {H}}}$ in order to evaluate the accuracy of the suggested forecasting methods. Results of the forecast were compared with the actual past demand. We calculated Mean Absolute Error (MAE) and Mean Squared Error (MSE) for each demand category. Average values of MAE and MSE across all categories are equal to 0.12 and 0.14 for M-Holt, 0.095 and 0.114 for M-Moving and 0.089 and 0.1 for M-Same, respectively, that is, they deviate less than 15% from the best value, which is zero.

Some reservations made in the past may be canceled in the future before their check-ins. In order to account for this situation, we calculate the probability of cancellation for each demand category and assign a random number between 0 and 1 to each realized reservation. If this random number is less than or equal to the corresponding probability of cancellation, then the reservation is not counted in the future. For a given demand category, the ratio between the number of canceled reservations and the total number of reservations in the historical period for this category is taken as the probability of cancellation. Reservations that have been made and decided to be counted in the future reduce the number of rooms available during the corresponding period of stay.

In times of the COVID-19 crisis, historical data are few, and hence, they are less meaningful. In this situation, we suggest to consider several future demand scenarios such as pessimistic, moderate and optimistic ones. We suggest that the moderate future demand is obtained based on the available data, and pessimistic and optimistic future demands are obtained by multiplying the moderate demand by certain coefficients.

## Demand-price relations

We restrictively assume that the demand is a linear non-increasing function of the price. Consider an arbitrary check-in day $\tau$ and an arbitrary eligible demand category $c\in C_\tau$. Assume that the demand for this category and day is a linear non-increasing function $f_{\tau ,c}$ of the price $p_{\tau ,c}$ for this day and category: $f_{\tau ,c}(p_{\tau ,c})=a_{\tau ,c}-b_c p_{\tau ,c}$, where $b_c>0$ is the category dependent slope of the demand function, which is also called *elasticity coefficient* in the demand-price studies, see Marshal (1890), and $a_{\tau ,c}>0$ is a constant. The elasticity coefficient tends to change only in a long term under the influence of external political and economical factors such as the COVID-19 crisis. Therefore, it does not depend on $\tau$ and it may be applied to all days in the planning horizon within the same category. Contrarily, the constant $a_{\tau ,c}$ of the demand function $f_{\tau ,c}$ depends both on the demand category *c* and the day $\tau$.

**Slope of the demand function**. The elasticity coefficient for the demand category *c* is determined by a simple linear regression approach. A maximal set of historical data of the category is employed to fit a straight line through the set of data points in such a way that makes the sum of squared residuals of the simple linear regression model as small as possible. The fitted line represents the historical demand function, and its slope determines the elasticity coefficient $b_c$. It is important to employ all historical data available in order to get more realistic estimate of the slope of the demand function. Causal (or *explanatory*) variable of the regression function is the room price adjusted for inflation, and dependent variable is the number of rooms sold for this price.

An excessive sparseness of the data of some categories and the influence of unpredicted factors may cause producing negative values of the elasticity coefficients $b_c$. Since the demand function $f_{\tau ,c}$ is assumed to be non-increasing, negative coefficients $b_c$ are considered as deviations from the law. In such a case, we force $b_c$ to take zero value. In the computer experiments with data of the hotel ${\mathcal {H}}$, no case with a negative coefficient $b_c$ was observed.

**Constant** $a_{\tau ,c}$. We suggest that the constant $a_{\tau ,c}$ is calculated as $a_{\tau ,c}={\hat{z}}_{\tau ,c}+b_cp^0_c$, where $p^0_c$ is the reference price for category *c* and ${\hat{z}}_{\tau ,c}$ is the forecasted number of occupied rooms for this category in day ${\tau }$. Let $h_c$ denote the operational cost of a room in category *c*. Since $p^0_c<h_c$ implies negative profit for room category *c*, we reasonably assume that $p^0_c\ge h_c$ for all *c*. As we already mentioned, the reference price can be periodically adjusted by the managers for prices of competitors.

## Optimization

Our optimization model aims at maximizing the total profit of the hotel. Input data for the model are the defined parameters $a_{\tau ,c}$ and $b_c$ for each demand function $f_{\tau ,c}$, $c\in C_\tau$, the lower and upper bounds $L_c$ and $U_c$ on the price values of each category *c*, obtained as 50% deviations from the reference price $p^0_c$, the room operational cost $h_c$ for each category *c*, and the number of available rooms $R_{\tau ,i}$ for demand categories of each tariff-convertible type *i* in each day $\tau$. Since $U_c<h_c$ implies negative profit for category *c*, we reasonably assume that $U_c\ge h_c$ for all *c*. Based on the price hierarchy assumption, we also reasonably assume that $p^0_c\le p^0_{c+1}$ and $h_c\le h_{c+1}$ for $c\in C_\tau \cap M_i$, $c+1\in C_\tau \cap M_i$, and any $\tau$ and *i*. Maximization of the total profit of a hotel is gained via solving the following quadratic programming problem with room prices $p_{\tau ,c}$ as the variables.

4 $$\begin{aligned} \max \ \ \ \sum _{\tau =t+1}^{t+T}\sum _{c\in C_\tau }(a_{\tau ,c}-b_c p_{\tau ,c})(p_{\tau ,c}-h_c), \end{aligned}$$

subject to

5 $$\begin{aligned}&L_c \le p_{\tau ,c}, \quad \tau =t+1,\ldots ,t+T, \; c\in C_\tau , \end{aligned}$$

6 $$\begin{aligned}&p_{\tau ,c} \le U_c, \quad \tau =t+1,\ldots ,t+T, \; c\in C_\tau , \end{aligned}$$

7 $$\begin{aligned}&a_{\tau ,c} \ge b_c p_{\tau ,c}, \quad \tau =t+1,\ldots ,t+T, \; c\in C_\tau , \end{aligned}$$

8 $$\begin{aligned}&p_{\tau ,c} \ge h_c, \quad \tau =t+1,\ldots ,t+T, \; c\in C_\tau , \end{aligned}$$

9 $$\begin{aligned}&\sum _{c\in M_i\cap C_\tau }(a_{\tau ,c}-b_c p_{\tau ,c}) \le R_{\tau ,i},\quad i=1,\ldots ,r, \; \tau =t+1,\ldots ,t+T, \end{aligned}$$

10 $$\begin{aligned}&p_{\tau ,c} \le p_{\tau ,c+1} , \quad c\in C_\tau \cap M_i, \; c+1\in C_\tau \cap M_i,\ i=1,\ldots ,r, \; \tau =t+1,\ldots ,t+T, \end{aligned}$$

11 $$\begin{aligned}&p_{\tau ,c}\ge 0, \quad \forall \ \tau ,\ c\in C_\tau . \end{aligned}$$

Relations (5) and (6) address price lower and upper bounds, respectively. Constraints (7) guarantee that the demand takes non-negative values. Relations (8) require that the room price is not less than the room operational cost. Constraints (9) ensure that the sum of the requested number of rooms in different categories of the same tariff-convertible type *i* and day $\tau$ does not exceed their available number $R_{\tau ,i}$. Constraints (10) secure the price hierarchy between categories of the same tariff-convertible type. Our optimization model does not allow overbooking. It may happen that the forecasted demand for the reference price exceeds the corresponding room capacity. Then the model will try to increase the price in order to decrease the demand, and by doing so, satisfy the capacity constraint.

Observe that, due to the assumptions about the input parameters, $L_c\le U_c$, $L_c\le p^0_c=\frac{a_{\tau ,c}-{\hat{z}}_{\tau ,c}}{b_c}\le \frac{a_{\tau ,c} }{b_c}$, $h_c\le p^0_c\le \frac{a_{\tau ,c} }{b_c}$ and $h_c\le U_c$ for $c\in C_\tau$. It follows that $\max \{L_c,h_c\}\le \min \{U_c,\frac{a_{\tau ,c} }{b_c}\}$ for all pairs $(\tau ,c)$ such that $c\in C_\tau$, and therefore, a feasible solution of the relations (4)–(9), (11) always exists. Regarding the relations (10), they are satisfied if and only if

12 $$\begin{aligned}&\max \{L_{c},h_{c}\}\le \min \left\{ U_{c+1},\frac{a_{\tau ,c+1} }{b_{c+1}}\right\} ,\quad c\in M_i\cap C_\tau ,\nonumber \\&\quad c+1\in M_i\cap C_\tau ,\ \tau =t+1,\ldots ,t+T,\ i=1,\ldots ,r. \end{aligned}$$

Since $L_c\le p^0_c\le p^0_{c+1}\le U_{c+1}$, $L_c\le p^0_c\le p^0_{c+1}\le \frac{a_{\tau ,c+1} }{b_{c+1}}$, $h_c\le h_{c+1}\le U_{c+1}$ and $h_c\le h_{c+1}\le p^0_{c+1}\le \frac{a_{\tau ,c+1} }{b_{c+1}}$, relations (12) are also satisfied.

We have shown that the problem (4)–(11) always has a feasible solution. It is a mathematical programming problem with a concave quadratic objective function and linear constraints. The problem can be decomposed into *T* subproblems. Each such a subproblem considers one day $\tau$, $\tau =t+1,\ldots ,t+T$. An optimal solution of the original problem is determined by the optimal solutions of the subproblems. The problem can be solved by a standard optimization software, for example, CPLEX or MATLAB.

Solving problem (4)–(11) specifies optimal price $p^*_{\tau ,c}$ for each category $c\in C_\tau$ in each day $\tau$ of the planning horizon. Data for the considered hotel ${{\mathcal {H}}}$ includes 5 room types, 2 values of the parameter *Length* and 3 values of the parameter *Before*. Therefore, the cardinality of the daily set of optimal prices for the hotel ${{\mathcal {H}}}$ is 30. If the period of stay of incoming reservation starts in one category (low season or weekday) and ends in another category (high season or weekend), then the period of stay is divided into several parts, each of a unique category, and the corresponding price is calculated for each part. Given optimal prices $p^*_{\tau ,c}$, the corresponding anticipated demands $a_{\tau ,c}-b_c p^*_{\tau ,c}$ can be computed. These values are estimates of the hotel occupancy for each day and room type and they can be used for planning service activities. A solution of the problem (4)–(11) can be analysed and approved or modified by the decision makers. An approved solution is made accessible to the potential guests of the hotel.

There can be two booking policies based on the solution of the problem. The first policy is to accept every incoming request and update the solution after each booking. The second policy is to accept as many requests from each category as determined by the optimal demand values $a_{\tau ,c}-b_c p^*_{\tau ,c}$. The excessive requests will be rejected. The efficiency of the second policy strongly depends on the demand forecast quality. Irrespectively of the booking policy, we suggest price update to occur after every realized booking, because every booking decreases the number of available rooms. We also suggest to set up several planning horizons with different lengths, for example for 1, 7, 31, 90, 180 and 360 days, and update solutions for all of them simultaneously. Accuracy of the forecast and, therefore, solution quality decreases as the length of the planning horizon increases. Therefore, solutions for the shorter planning horizon should be more accurate.

## Computer experiments

In order to evaluate the efficiency of our dynamic pricing approach, we conducted computer experiments to compare the actual revenue of the example hotel ${{\mathcal {H}}}$ in the past period with the modeled potential revenue generated by our approach for the same past period, which we call *comparison period*, subject to the assumption that any demand value in this period is randomly drawn from the interval [0.95*d*, 1.05*d*], where *d* is the demand value calculated according to the elastic model described in Sect. 5.

In the experiments, we use data of the hotel ${{\mathcal {H}}}$ disturbed because of the confidentiality. The room operational cost, $h_c$, is set to 50 for all categories. Reference prices of the most valuable categories of tariff classes *E*, *E+*, *B*, *B+* and *S* are set to 139, 149, 159, 175 and 189, respectively. Percentage deviations from these reference prices for categories with parameters *Season=Low*, *Day=Mon–Thu*, *Length=8+*, *Before=8–30* and *Before=30+* are set to 20%, 10%, 10%, 10% and 15%, respectively. The approach employs the M-Moving forecasting method.

A past period of 90 days is considered. It is divided by a fixed day *t* into two periods: initial historical period of 30 days $t-29,t-28,\ldots ,t$, and initial planning horizon of 60 days $t+1,t+2,\ldots ,t+60$. 14 days $t+31,t+32,\ldots ,t+44$ are used as the comparison period. The reason for choosing these days is that no booking with parameter *Before=31+* that have been made in day *t* or after can have a check-in in day $t+30$ or before.

The experiments were run on a PC with Intel Core i5 $2.4\times 2$ GHz processor and 4 GB of RAM under MS Windows 8.1 Pro (64 bit). Three types of scenarios have been considered for the selection of the historical input data. In the *steady* scenario, daily number of check-ins in the historical period does not differ much from the daily number of check-ins in the planning horizon. *Low-grow* and *high-grow* scenarios are characterized by low and high growth, respectively, of daily check-ins in the planning horizon in comparison with the same values in the historical period.

In each scenario, the experiment was run 14 times, once for each $\tau =t,t+1,\ldots ,t+13$. In each run, the solution method was applied for the historical period $\tau -29,\tau -28,\ldots ,\tau$ and the planning horizon $\tau +1,\tau +2,\ldots ,\tau +60$. The modeled revenue was calculated for the day $\tau +31$ of the comparison period. Numerical results are shown in Table 1.

**Table 1:** Comparison of revenues

Low-grow scenario			High-grow scenario			Steady scenario		
Day #	Fixed-price revenue	Dynamic pricing revenue	Day #	Fixed-price revenue	Dynamic pricing revenue	Day #	Fixed-price revenue	Dynamic pricing revenue
1	142.5	144.6	1	0	62.9	1	0	50.8
2	0	62.4	2	285.0	262.2	2	527.5	561.0
3	0	26.3	3	0	49.0	3	325	367.1
4	487.5	502.1	4	142.5	190.9	4	152.5	208.3
5	782.5	743.2	5	557.5	604.1	5	822.5	788
6	1907.5	1682.2	6	1167.5	974.3	6	162.5	233.4
7	507.5	774.7	7	0	72.8	7	842.5	812.1
8	477.5	701.8	8	325	235.2	8	822.5	790.3
9	0	45.2	9	152.5	249.2	9	822.5	792.2
10	0	56.2	10	152.5	237.3	10	182.5	215.7
11	0	78.4	11	162.5	212.2	11	0	297.4
12	0	93.6	12	945	910.3	12	0	53.6
13	3025	2834.6	13	872.5	831.8	13	0	34.5
14	1270	1168.4	14	0	63.9	14	152.5	98.8
Total	8600.0	8913.1		4762.5	4956.1		4812.5	5303.2
**Growth**		**3.6%**					**4.1%**	**10.2%**
**Average growth**								**5.97%**

Experiments demonstrate that our dynamic pricing approach increases the average revenue of the hotel in comparison with the static pricing strategy. Moreover, due to the fact that the cost structure of the hotel business is characterized by high fixed and low variable costs, the ability of the approach to increase revenue brings even relatively greater contribution to the profit than to the revenue.

## Conclusions

We describe a dynamic pricing approach for a hotel revenue management problem with multi-products. The demand is divided into several categories, the forecast is made for each category and the optimal prices for the categories are found by solving a mathematical programming problem with a concave quadratic objective function and linear constraints. As soon as the optimal prices have been determined, the demand function can be used to determine the number of rooms that should be assigned to each category. One decision strategy is to accept any booking in a category if a suitable room is available. Another strategy is to only accept the number of bookings determined by the demand function and optimal prices. Our approach can take into account the prices of competitors as these are included in the reference prices of the demand categories. The approach can be used to plan prices for the period up to a year in advance. It can be used to manage a single hotel or a hotel chain, provided that the season periods of all hotels in the chain are the same.

The special features of our approach are:

- Handling multiple products;
- Addressing the lengths of stays;
- Addressing the hotel capacity;
- The flexibility of shifting a room from one tariff class into a different tariff class;
- No limits on the number of price changes;
- The planning horizon is up to a year;
- The demand-price function allows a short-term modeling in times of a crisis like COVID-19 pandemic.

Computer experiments have shown that the suggested dynamic pricing approach can increase hotel revenues. The approach is expected to be more efficient in terms of the computational resources than the existing stochastic programming, dynamic programming and fuzzy goal programming methods adapted for hotel dynamic pricing and revenue management.

During the COVID-19 crisis hotels need to be reactive and flexible. The suggested revenue management tool allows to audit reservations and calculate different future demand scenarios. The choice of the hotel for a traveller is based on several factors such as long period reduction, room upgrades, high season offers, special cancellation offers, etc., which can be accounted in the demand categories. The tool offers the hotel business the opportunities of an improved price management for various scenarios and a quick reaction on market and demand changes. It can support the recovery of small hotels from the economic consequences of COVID-19.

Note that a long employment of the dynamic pricing approach can lead to an *endogeneity* problem in determining correct demand-price relations. The problem is that the hotel changes prices in response to demand and demand changes in response to price. In this case, the suggested simple linear regression model can produce lower values of the elasticity coefficient than its true values, that is, the price elasticity can be underestimated. Further research is required to address the endogeneity problem if it does arise.
